# Mutational screening of the *USH2A *gene in Spanish USH patients reveals 23 novel pathogenic mutations

**DOI:** 10.1186/1750-1172-6-65

**Published:** 2011-10-17

**Authors:** Gema Garcia-Garcia, Maria J Aparisi, Teresa Jaijo, Regina Rodrigo, Ana M Leon, Almudena Avila-Fernandez, Fiona Blanco-Kelly, Sara Bernal, Rafael Navarro, Manuel Diaz-Llopis, Montserrat Baiget, Carmen Ayuso, Jose M Millan, Elena Aller

**Affiliations:** 1 Grupo de Investigación en Enfermedades Neurosensoriales. Instituto de Investigación Sanitaria IIS-La Fe, Valencia, Spain; 2 CIBER de Enfermedades Raras (CIBERER), Valencia, Spain; 3 Servicio de Genética, Fundación Jiménez Díaz, Madrid, Spain; 4 Servei de Genètica, Hospital de la Santa Creu i Sant Pau, Barcelona, Spain; 5 Instituto de Microcirugía Ocular, Barcelona, Spain; 6 Servicio de Oftalmología, Hospital Universitario La Fe, Valencia, Spain; 7 Unidad de Genética y Diagnóstico Prenatal, Hospital Universitario La Fe, Valencia, Spain

**Keywords:** Usher Syndrome, *USH2A*, Mutations, Sequence Variants

## Abstract

**Background:**

Usher Syndrome type II (USH2) is an autosomal recessive disorder, characterized by moderate to severe hearing impairment and retinitis pigmentosa (RP). Among the three genes implicated, mutations in the *USH2A *gene account for 74-90% of the USH2 cases.

**Methods:**

To identify the genetic cause of the disease and determine the frequency of *USH2A *mutations in a cohort of 88 unrelated USH Spanish patients, we carried out a mutation screening of the 72 coding exons of this gene by direct sequencing. Moreover, we performed functional minigene studies for those changes that were predicted to affect splicing.

**Results:**

As a result, a total of 144 DNA sequence variants were identified. Based upon previous studies, allele frequencies, segregation analysis, bioinformatics' predictions and *in vitro *experiments, 37 variants (23 of them novel) were classified as pathogenic mutations.

**Conclusions:**

This report provide a wide spectrum of *USH2A *mutations and clinical features, including atypical Usher syndrome phenotypes resembling Usher syndrome type I. Considering only the patients clearly diagnosed with Usher syndrome type II, and results obtained in this and previous studies, we can state that mutations in *USH2A *are responsible for 76.1% of USH2 disease in patients of Spanish origin.

## Disease Name and Definition

### Usher syndrome

Usher syndrome (USH) is an autosomal recessive disease characterized by hearing loss, retinitis pigmentosa (RP), and, in some cases, vestibular dysfunction. It is clinically and genetically heterogeneous and is the most common cause underlying deafness and blindness of genetic origin. Clinically, USH is divided into three types. Usher type I (USH1) is the most severe form and is characterized by severe to profound congenital deafness, vestibular areflexia, and prepubertal onset of progressive RP. Type II (USH2) displays moderate to severe hearing loss, absence of vestibular dysfunction, and later onset of retinal degeneration. Type III (USH3) shows progressive postlingual hearing loss, variable onset of RP, and variable vestibular response. To date, five USH1 genes have been identified: *MYO7A *(USH1B), *CDH23 *(USH1D), *PCDH15 *(USH1F), *USH1C*(USH1C), and *USH1G*(USH1G). Three genes are involved in USH2, namely, *USH2A *(USH2A), *GPR98 *(USH2C), and *DFNB31 *(USH2D). USH3 is rare except in certain populations, and the gene responsible for this type is *USH3A*.

## Background

Usher syndrome (USH) is an autosomal recessive disease characterized by the association of hearing loss and visual impairment due to retinitis pigmentosa (RP), with or without vestibular dysfunction. It is the most frequent cause of concurrent deafness and blindness of genetic origin and its general prevalence ranges from 3.3 to 6.4 per 100.000 live births [1]. In Spain, the estimation is 4.2/100.000 [2].

USH is clinically and genetically heterogeneous. Three clinical forms are distinguished: USH1, USH2 and USH3 and nine genes have been identified responsible so far. Five causative genes have been reported for USH1: *MYO7A*, *USH1C*, *CDH23*, *PCDH15 *and *USH1G*. Three genes for USH2: *USH2A*, *GPR98 *and *DFNB31*. Meanwhile only one gene has been described for USH3: *USH3A *[3,4].

USH2 appears to be the most common clinical form of the disorder, accounting for more than 50% of all Usher cases [5,6]. Among the three genes described for USH2, *USH2A *is the most commonly mutated gene. It is responsible for approximately 74-90% of USH2 cases [2,7]. Mutations in *USH2A*, are also responsible for atypical Usher syndrome and recessive non-syndromic RP [8,9]. The *USH2A *gene, located on chromosome 1q41 [10], was initially described as comprising 21 exons, encoding a protein of 1546 amino acids [11,12]. However, in 2004, van Wijk et al. (2004) identified 51 additional exons at the 3' end of *USH2A *[13]. The longest transcript consists of 72 exons, encoding a protein of 5202 amino acids. In addition, Adato et al. (2005), identified an alternative spliced exon 71 in mouse transcripts, expressed in the inner ear and well conserved in vertebrates [14]. The long *isoform b *is characterized by containing a transmembrane region, followed by an intracellular domain with a PDZ-binding motif, which interacts with the PDZ domain of harmonin and whirlin, integrating USH2A into the USH protein network [14,15].

Initially, studies of the *USH2A *gene covered just exons 2-21 [11,16,8,12,9,22], but only 45-63% of the expected *USH2A *mutations were identified. Nevertheless, since the discovery of the long isoform, several mutational analyses of all 72 exons have been carried out in diverse populations [23-30]. As a result, many novel pathogenic mutations have been identified, including splicing mutations at non-canonical positions of splice sites [31]. However, the majority of these changes only appear in a few cases, with the exception of the common ancestral p.Glu767fs mutation, located at exon 13, which is the most prevalent *USH2A *mutation in several populations [16].

In the present study we have performed an exhaustive mutational screening of the long *isoform b *of *USH2A *to identify new patients with mutations in this gene, and to detect the second mutation in patients with one previously detected *USH2A *mutation. Some cases had previously been studied for exon 13 or for the 21 first *USH2A *exons [19,9], or analyzed using the genotyping microarray for Usher syndrome (Asper Biotech, Tartu, Estonia; [32]). Furthermore, we have used *in silico *and *in vitro *analysis to evaluate the functional consequences on gene expression and protein function of several nucleotide changes.

## Materials and methods

### Subjects

Eighty-eight (88) unrelated Spanish patients diagnosed of Usher syndrome were included in this study. They were recruited from the *Federación de Afectados de Retinosis Pigmentaria de España *(FARPE) and also from the Ophthalmology and ENT Services of several Spanish Hospitals as part of a large-scale study on the genetics of Usher syndrome in the Spanish population.

On the basis of their clinical history and ophthalmologic, audiological, neurophysiological and vestibular tests, 58 of these families were clinically classified as USH2 while 11 displayed atypical Usher syndrome. Detailed clinical data could not be obtained for 19 patients and these remained as non classified (USHNC).

Previously, 40 of these 88 patients were studied for exon 13, while 24 were analysed for the first 21 exons of *USH2A *and 42 were analyzed with the genotyping microarray for Usher syndrome (Asper Biotech, Tartu, Estonia). At that time, the version of the array detected 429 previously described mutations in eight of the nine genes reported for the disease. As a result of these previous analyses, eighteen of them were found to carry one mutated allele, but the second mutation could not be detected. These mutations have been included in the statistical summaries presented herein. These patients were subjected to mutation screening of the exons that had not been analyzed. In the remaining patients, we carried out the study of exons 2-72 (including the alternatively spliced exon 71).

When DNA samples from patients' relatives were available, we carried out a segregation analysis.

One hundred unrelated individuals of Spanish origin without hearing loss or RP family history were screened as controls to evaluate the frequency of the mutations found in the patient sample.

### Mutation analysis

Genomic DNA from patients and controls was extracted from peripheral blood samples following standard protocols. The coding exons and flanking intronic sequences of *USH2A *were amplified by PCR using primers and conditions described by Aller et al. (2004; 2006) [9,23]. The amplified DNA fragments were analysed by direct sequencing using the Big Dye Terminator v.3.1 kit *(*Applied Byosistems, Carlsbad, CA*)*, and purified sequencing reactions were analysed in an ABI PRISM 3730 DNA analyzer *(*Applied Byosistems, Carlsbad, CA). The obtained sequences were compared with the consensus sequence NM_206933.2. The +1 position corresponds to A in the ATG translation initiation codon.

### Predictions of the pathogenic effect of missense variations

To predict whether a rare missense variant is deleterious, we used the combined results of three different computer algorithms:

-Sort Intolerant From Tolerant (SIFT) (available at http://sift.jcvi.org) uses sequence homology to predict whether a change is tolerated or deleterious.

-The polymorphism phenotyping program, PolyPhen (available at http://genetics.bwh.harvard.edu/pph/) uses sequence conservation, structure and SWISS-PROT annotation to characterize an amino acid substitution as benign, possibly deleterious or probably deleterious.

-Pmut (available at http://mmb2.pcb.ub.es:8080/PMut/) provides prediction by neural networks, which use internal databases, secondary structure prediction and sequence conservation. This program provides a binary prediction of "neutral" or "pathologic".

### Splice-site Prediction programs

Intronic, isocoding and missense changes were analyzed using the programs NNSPLICE (http://fruitfly.org:9005/seq_tools/splice.html), Human Splicing Finder (HSF) version 2.4 (http://www.umd.be/HSF/) and NetGene2 (http://www.cbs.dtu.dk/services/NetGene2/) in order to predict whether those changes could be affecting, creating or eliminating donor/acceptor splice sites.

### Minigene constructions and expression

Minigene constructs were generated, using the exon trapping expression vector pSPL3. For each mutation, the exon and intronic flanking sequences were amplified from the patient's DNA, using the High Fidelity Phusion polymerase (Finnzymes, Espoo, Finland). Amplicons were inserted between the XhoI/NheI and XhoI/BamHI restriction sites for the variants p.E2496E and p.V382M, respectively, using T4 DNA ligase (Invitrogen Corporation, Carlsbad, CA). The p.V382M mutation was generated by site-directed mutagenesis. All vectors were confirmed by direct sequencing. The minigene constructs were transfected into COS-7 cells as described before [33]. RNA extraction and RT-PCR analysis was perfomed as previously described [34,31]. Missplicing percentages were measured using the Alpha Imager 2200 (version 3.1.2) software (AlphaInnotech Corporation, San Francisco, CA, USA).

## Results

The molecular analysis of the *USH2A *gene in 88 unrelated USH Spanish patients revealed 37 different pathogenic mutations. Among these, a total of 23 mutations were novel (See Tables 1, 2 and 3). At least one pathogenic mutation was found in 43 out of 88 unrelated patients (48.9%). Thirty-three patients were classified as USH2, five as USHA (atypical Usher syndrome) and five as USHNC (Usher syndrome non classified). In 25 out of these 43 cases the two causative mutations were detected (58.1%), five patients were homozygous and 20 compound heterozygous. Detailed clinical manifestations of these 25 patients and 3 additional patients with one pathogenic and one probably pathogenic mutation (UV3; likely to be pathogenic but cannot formally be proven) are summarized in Table 4.

**Table 1:** *USH2A *truncating and *splice-site *mutations

Nucleotide change	Exon	Predicted effect	Predicted pathology	No. of alleles	References
**Nonsense mutations**					

**c.820C > T**	5	**p.R274X**	+	2	Present study

c.1518T > A	8	p.Y506X	+	1	Bernal *et al*., 2005

c.3883C > T	18	p.R1295X	+	1	Dreyer *et al*., 2000

c.4474G > T	21	p.E1492X	+	2	Bernal *et al*., 2005

c.4645C > T	22	p.R1549X	+	1	Baux *et al*., 2007

**c.7854G > C**	41	**p.W2618X**	+	1	Present study

**c.9753T > A**	50	**p.C3251X**	+	2	Present study

**c.10102C > T**	51	**p.Q3368X**^**a**^	+	1	Present study

c.10759C > T	55	p.Q3587X	+	2	Baux *et al*., 2007

**c.11146C > T**	57	**p.Q3716X**	+	1	Present study

**c.14175G > A**	65	**P.W4725X**	+	1	Present study

**Deletions and insertions**					

**c.918_919insGCTG**	6	**p.S307AfsX17**	+	1	Present study

c.1214delA	7	p.N405IfsX3	+	5	Bernal *et al*., 2005

**c.1629_1645del**	10	**p.F543LfsX2**	+	1	Present study

c.2299delG	13	p.E767SfsX21	+	8	Eudy *et al.*, 1998

**c.5278delG**	26	**p.D1760MfsX10**^**a**^	+	1	Present study

**c.5540_5541dup**	27	**p.N1848X**	+	1	Present study

**c.6319_6324delinsTAAA**	32	**p.V2107X**	+	1	Present study

**c.8890dupT**	45	**p.W2964LfsX89**	+	1	Present study

**c.8954delG**	45	**p.G2985AfsX3**	+	1	Present study

**c.9261delT**	47	**p.E3088KfsX9**	+	1	Present study

c.10272_10273dupTT	52	p.C3425FfsX4	+	1	Aller *et al.*, 2006

**c.11566delA**	60	**p.S3856VfsX28**	+	1	Present study

**c.12093delC**	62	**p.Y4031X**	+	1	Present study

**c.13140delA**	63	**p.V4381CfsX10**	+	1	Present study

***Splice-site *mutations**					

**c.1328 + 1G > T**	IVS7	Ex7 splice defect	+	1	Present study

c.1841-2A > G	IVS10	Ex11 splice defect	+	2	Bernal *et al*., 2003

**c.11548 + 2T > G**	IVS59	Ex59 splice defect	+	1	Present study

c.12067-2A > G	IVS61	Ex62 splice defect	+	4	Auslender *et al.*, 2008

**c.15053-1G > A**	IVS69	Ex70 splice defect	+	1	Present study

**+**: Denotes pathogenic mutations.

**No. of alleles**: Number of alleles identified in patients.

^a^These two mutations are allelic.

Novel pathogenic mutations described in this study are in bold.

**Table 2:** Missense changes in *USH2A*

Nucleotide change	Exon	Amino acid change	Predicted pathology	No. of alleles	**Ref**.
c.373G > A	2	p.A125T	-	81	Dreyer *et al*., 2000

**c.130G > A**	2	**p.G44R**	+	1	Present Study

c.688G > A	4	p.V230M	-	3	Dreyer *et al*., 2000

c.908G > A	6	p.R303H^e^	UV3	1	Yan *et al*., 2009

**c.1144G > A**	7	**p.V382M**^**b**^	UV3	1	Present Study

c.1434G > C	8	p.E478D	-	4	Seyedahmadi *et al*., 2004

c.1663C > G	10	p.L555V	-	2	Bernal *et al*., 2003

c.1931A > T	11	p.D644V	-	6	Weston *et al*., 2000

c.2137G > C	12	P.G713R	UV2	2	Dreyer *et al*., 2000

c.2276G > T	13	p.C759F	+	3	Dreyer *et al*., 2000

c.2522C > A	13	p.S841Y	UV2	1	Jaijo *et al*., 2009

c.4457G > A	21	p.R1486K	-	75	Dreyer *et al*., 2000

c.4714C > T	22	p.L1572F	-	7	Dreyer *et al*., 2008

c.4994T > C	25	p.I1665T	-	31	Kaiserman *et al*., 2007

c.5975A > G	30	p.Y1992C^e^	UV3	1	McGee et al., 2010

c.6317T > C	32	p.I2106T	-	117	Aller *et al*., 2006

c.6506T > C	34	p.I2169T	-	85	Aller *et al*., 2006

c.6587G > C	34	p.S2196T	UV2	2	Jaijo *et al*., 2009

c.6713A > C	35	p.E2238A	-	1	Aller *et al*., 2006

c.6875G > A	36	p.R2292H	-	2	Dreyer *et al*., 2008

c.7130A > G	38	p.N2377S	UV2	1	Present Study

c.7182C > A	38	p.N2394K	UV2	1	Present Study

c.7506G > A	40	p.P2502P	-	11	Baux *et al*., 2008

c.7685T > C	41	p.V2562A	-	1	Dreyer *et al*., 2008

c.7915T > C	41	p.S2639P	UV2	3	McGee et al., 2010

c.8624G > A	43	p.R2875Q	-	9	Aller *et al*., 2006

c.8656C > T	43	p.L2886F	-	9	Aller *et al*., 2006

c.9262G > A	47	p.E3088K	-	1	Dreyer *et al*., 2008

c.9296A > G	47	p.N3099S	-	12	Aller *et al*., 2006

c.9343A > G	47	p.T3115A	-	9	Dreyer *et al*., 2008

c.9430G > A	48	p.D3144N	-	8	Aller *et al*., 2006

c.9595A > G	49	p.N3199D	-	9	Baux *et al*., 2007

c.9799T > C	50	p.C3267R	+	5	Aller *et al*., 2006

c.10073G > A	51	p.C3358Y	+	1	McGee *et al*., 2010

c.10232A > C	52	p.E3411A	-	94	Aller *et al*., 2006

**c.10636G > A**	54	**p.G3546R**	+	4	Present Study

c.11504C > T	59	p.T3835I	-	30	Present Study

c.11602A > G	60	p.M3868V	-	34	Aller *et al*., 2006

c.11677C > A	60	p.P3893T	-	2	Dreyer *et al*., 2008

**c.11680A > G**	60	**p.N3894D**	UV3	1	Present Study

c.12343C > T	63	p.R4115C	-	2	van Wijk *et al*., 2004

c.14074G > A	64	p.G4692R^c^	UV2	1	McGee *et al*., 2010

c.14453C > T	66	p.P4818L	+	1	Aller *et al*., 2006

c.14513G > A	66	p.G4838E	-	1	McGee *et al*., 2010

c.14543G > A	66	p.R4848Q	-	1	McGee *et al*., 2010

c.14761G > A	67	p.E4921K	UV2	1	Present Study

c.15076A > G	70	p.K5026E	UV2	1	McGee *et al*., 2010

c.15091C > T	70	p.R5031W	-	2	Dreyer *et al*., 2008

**+**: Denotes pathogenic mutations; **UV3**: Probably pathogenic mutations; **UV2**: probably non- pathogenic mutations; - : neutral varirants.

**No. of alleles**: Number of alleles identified in patients.

^b^These variant may alter normal splicing.

^c^Patient with this change also has two other clearly pathogenic mutations in *USH2A*.

^e ^p.R303H and p.Y1992C were initially described as being pathogenic mutations by Yan *et al*., 2009 (ref. 29) and McGee et al., 2010 (ref. 30) respectively, but we have classified them as UV3 in accordance with the specific locus database for Usher syndrome: https://grenada.lumc.nl/LOVD2/Usher_montpellier

Novel pathogenic mutations and novel probably pathogenic mutations (UV3) described in this study are in bold.

**Table 3:** Silent variants in *USH2A*

Nucleotide change	Exon	Amino acid position	Predicted pathology	No. of alleles	**Ref**.
c.504A > G	3	p.T168T	-	75	Baux *et al*., 2008

c.1179A > G	7	p.Q393Q	-	1	Aller, 2008

c.1419C > T	8	p.T473T	-	36	Dreyer *et al*., 2000

c.2109T > C	12	p.D703D	-	7	Weston *et al*., 2000

c.2256T > C	13	p.H752H	-	3	Dreyer *et al*., 2008

c.3945T > C	18	p.N1315N	UV2	3	Present Study

c.4371G > A	20	p.S1457S	-	1	Dreyer *et al*., 2000

c.5031C > A	25	p.G1671G	-	22	Aller *et al*., 2006

c.5751C > T	28	p.Y1917Y	UV2	1	McGee *et al*., 2010

**c.7488A > G**	40	**p.E2496E**^**b**^	+	1	Present Study

c.7506G > A	40	p.P2502P	-	11	McGee *et al*., 2010

c.11736G > A	61	p.E3912E	-	2	Dreyer *et al*., 2008

c.11907A > T	61	p.P3969P	-	2	Dreyer *et al*., 2008

c.11946A > T	61	p.L3982L	-	29	Dreyer *et al*., 2008

c.12093C > T	62	p.Y4031Y	-	1	Dreyer *et al*., 2008

c.12612A > G	63	p.T4204T	-	129	Dreyer *et al.*, 2008

c.12666A > G	63	p.T4222T	-	58	Dreyer *et al*., 2008

c.13191G > A	63	p.E4397E	-	23	Dreyer *et al*., 2008

c.14481C > T	66	p.A4827A	-	1	McGee *et al*., 2010

c.14664G > A	67	p.T4888T	UV2	1	Present Study

**+**: Denotes pathogenic mutations; **UV2**: probably non- pathogenic mutations; - : neutral variants.

**No. of alleles**: Number of alleles identified in patients.

^b^These variant may alter normal splicing.

^c^Patient with this change also has two other clearly pathogenic mutations in *USH2A*.

Novel pathogenic mutations described in this study are in bold.

**Table 4:** Genotype-phenotype correlations of USH patients with both mutations found in this study

Patient	Mutations	Year of Birth	Diagnosis	Age of diagnosis	Sensorineural Hearing Loss	Vestibular Function	Onset of Night Blindness	Onset of Visual Field Loss	Visual Field	Visual Acuity	Eye Fundus	ERG	Cataracts
RP1310*	**c.12067-2A > G/c.12067-2A > G**		**USHNC**										

RP1274*	p.E1492X/p.E1492X	1969	USH2		Moderate-severe and stable	Normal^A^					1		

RP1633	p.E1492X/p.E1492X	1962	USH2	25	Since infancy	Normal^A^	25	25	Concentric loss	0,2/0,4	1	No response	BE

RP1607	p.G3546R/p.G3546R	1925	USH2	33	Mild-moderate	Normal^A^	16	25	Marked concentric loss	0,6/0,6	1	Moderate alteration	BE

RP1599*	c.1214delA/c.1214delA	1980	USH2		Moderate, since infancy	Normal^A^	20	30	Moderate concentric loss	0,6/0,7	1	Moderate alteration	BE

**RP259**	**c.1214delA/p.C3267R**	**1974**	**USHA**	**19**	**Profound since 6 years**	**Vestibular Dysfunction**^**A**^	**6**		**Concentric loss (19 years)**	**0,35/0,35 (19 years)**	**1**	**No response**	**No (19 years)**

RP1349	c.2299delG/c.1214delA	1954	USH2		Moderate-severe	Normal^B^	15	20	Marked concentric loss	0,4/0,3 (30 years)	2		

RP1493	c.2299delG/c.8890dupT	1973	USH2	30	Congenital, moderate and stable	Normal^B^	29	29	Concentric loss (at 31 years)	Normal (31 years)	1	No response (31 years)	No (31 years)

RP1632	c.2299delG/c.8954delG	1961	USH2		Since infancy	Normal^B^	23	25		<0,1BE	2	No response	BE

RP1715*	c.2299delG/c.1629_1645del	1986	USH2	22	Moderate and stable since 6 years	Normal^B^	16	20	Slight concentric loss (at 25 years)	Normal (25 years)	1	No response	No

**RP1775**	**c.2299delG/p.R303H**^**#**^	**1961**	**USHA**	**30**	**Moderate since 7 years and progressive**		**23**	**22**					

RP1618	p.C3267R/c.6319_6324delinsTAAA	1964	USH2		Severe-profound	Normal^A^	30	30	Marked concentric loss	0,1/0,1	2	No response	BE

RP1442*	p.C3267R/c.12093delC	1962	USH2	20	Congenital, moderate and stable	Normal^B^	15	20	Concentric loss, 5° (at 43 years)	0,5/0,2 (43 years)	2		LE (37 years) RE (43 years)

RP1703*	**p.C3267R/p.C3358Y**	**1936**	**USHA**	**50**	**Since 64 years**		**50**	**55**	**Reduced (67 years)**		**1**		**Yes**

RP1759	p.C3267R/p.Y1992C^#^	1945	USH2	62	Congenital, moderate and stable	Normal^B^		62				Abnormal response	Yes (62 years)

RP1625*	c.12067-2A > G/p.R274X	1976	USH2	25	Since infancy	Normal^A^	19	24	Concentric loss	0,1/0,1	2	No response	BE

RP1631	c.1841-2A > G/p.R274X	1976	USH2		Since infancy	Normal^A^	28	28	Concentric loss	0,7/0,6	1		No

RP951	c.1214delA/p.C3251X	1969	USH2	25	Congenital, severe and stable	Normal^B^	25	18	Reduced (23 years)		2	No response (30 years)	No (30 years)

RP1558	p.R1549X/c.1328 + 1G > T	1933	USH2	43	Severe and progressive since 20 years	Normal^B^	Before puberty		-10°C	0,007/0.03	2		

RP1539*	p.R1295X/p.N3894D^#^	1987	USH2	20	Congenital, moderate and stable	Normal^B^	20	17	Concentric loss (20 years)		2		

RP1172*	c.10272_10273dupTT/p.W2618X	1964	USH2	23	Moderate and stable since 7 years	Normal^B^	23	18	Concentric loss (38 years)			No response (39 years)	

RP1641*	**p.C759F/p.W4725X**	**1967**	**USHA**		**Moderate and progressive**	**Central vestibular pathology**^**A**^	**18**	**22**	**Marked concentric loss**	**0,1/0,2**	**2**	**No response**	**BE**

RP1667*	p.C759F/c.11548 + 2T > G	1954	USH2	15	Mild and slightly progressive	Normal^B^	15	8					RE (30 years)

RP690M*	p.P4818L/p.Q3368X + c.5278delG	1972	USH2	22	Congenital, moderate and stable	Normal^A^	22	8		0,3/0,3 (32 years)	1	No response (31 years)	BE (25 years)

RP532	p.Y506X/p.Q3587X	1968	USH2		Profound	Normal^A^	15	20	Marked concentric loss	0,1/0,1	2	No response	BE

RP946*	**p.Q3587X/p.E2496E**	**1982**	**USHNC**	**17**									

RP1613	c.9260delT/p.G44R	1971	USH2	30	Severe	Normal^A^	20	20	Marked concentric loss	0,1/0,6	2	Abnormal response	BE

RP1615	c.11566delA/c.15053-1G > A	1975	USH2	27	Moderate since infancy	Normal^A^	24	25	Marked concentric loss	0,5/0,5	2	No response	BE

*Parental origin of the mutations was determined; ^#^Patients with possibly pathogenic mutations (UV3).

Age of diagnosis, onset of night blindness and visual field loss are expressed in years.

^A^: results of clinical examinations; ^B^: self reported symptoms.

Eye fundus 1: Bone spicules deposits, attenuation of vessels and waxy pollar of the optic nerve head. Eye fundus 2: 1 + macular affectation.

ERG: Electroretinography; BE: Both Eyes; LE: Left Eye; RE: Right Eye.

Patients clinically classified as USHA or USHNC are highlighted in bold.

In this study, a total of 144 variants were detected: 25 were truncating mutations and five were splice-site mutations (located at the conserved AG/GT dinucleotides of the splice site). The pathogenic effect of these variants is clear. But, in addition, 48 missense, 20 silent and 46 intronic variants were identified. According to previous studies, allele frequencies, segregation analysis, bioinformatics' predictions and *in vitro *experiments, the missense, silent and intronic changes were classified into 4 different categories: pathogenic, possibly pathogenic (UV3), possibly non-pathogenic (possibly neutral, UV2) and non-pathogenic (neutral). (See Tables 1, 2,3 and 5).

**Table 5:** Novel intronic variants

Nucleotide change	Intron	Predicted pathology	No. of alleles
c.1328 + 52T > C	IVS7	-	3

c.1841-61G > A	IVS10	-	11

c.4627 + 32G > T	IVS21	UV2	1

c.6485 + 18C > T	IVS33	UV2	1

c.6486-54T > C	IVS33	UV2	2

c.6486-43T > A	IVS33	UV2	1

c.6657 + 29C > A	IVS34	UV2	1

c.8681 + 18A > G	IVS43	UV2	1

c.8681 + 53T > G	IVS43	UV2	1

c.8681 + 118A > G	IVS43	UV2	1

c.9056-52G > T	IVS45	UV	1

c.9372-50A > G	IVS47	UV2	1

c.9740-59G > A	IVS49	UV2	1

c.9958 + 128A > G	IVS50	-	4

c.10388-123T > C	IVS52	UV2	1

c.13812-78A > G	IVS63	UV2	1

c.14134-53T > C	IVS64	UV2	1

c.14343 + 36G > C	IVS65	UV2	1

c.15298-35T > A	IVS70	UV2	1

c.15298-1153G > A(g.798209G > A) ^d^	IVS71	UV2	1

**UV2**: probably non- pathogenic mutations; - : neutral variants.

**No. of alleles**: Number of alleles identified in patients.

^d^The intronic variant g.798209G > A affects the last nucleotide of the cochlea specific exon 71. g.DNA numbering starts at nucleotide position 1 in Human Refseq: NG_009497.1 GI:222352133, which represents the minus (-) strand of *USH2A*.

### Missense variants

Fourty-eight missense variants were identified (See Table 2). Twenty-nine were considered as non-pathogenic because all of them were already described as non-pathogenic in other studies [https://grenada.lumc.nl/LOVD2/Usher_montpellier, [35]].

Nine nucleotide changes were classified as possibly non-pathologic (UV2). p.G713R, p.S841Y, p.S2196T, p.S2639P, p.G4692R and p.K5026E have already been reported in other works and categorized as possibly non-deleterious or of unknown pathogenecity [https://grenada.lumc.nl/LOVD2/Usher_montpellier, [35]]. Meanwhile, p.N2377S, p.N2394K and p.E4921K have not been described previously, so they were analyzed with the three sequence analysis programs (SIFT, Polyphen and PMUT). None of those changes was predicted to be clearly deleterious (See Table 6).

**Table 6:** Results from the three different analysis programs used to predict the pathogenicity of novel missense changes

	SIFT	PolyPhen	PMUT
**p.G44R**	Affect (Score 0.01)	Probably damaging	Pathogenic (NN output 0.5113)

**p.N2377S**	Tolerated (Score 0.44)	Possibly damaging	Neutral (NN output 0.2372)

**p.N2394K**	Tolerated (Score 0.23)	Possibly damaging	Neutral (NN output 0.5113)

**p.G3546R**	Affect (Score 0.01)	Probably damaging	Pathogenic (NN output 0.1799)

**p.N3894D**	Tolerated (Score 0.05)	Probably damaging	Neutral (NN output 0.0522)

**p.E4921K**	Tolerated (Score 0.85)	Benign	Neutral (NN output 0.3130)

**SIFT**: SIFT Score ranges from 0 to 1. The amino acid substitution is predicted to be damaging if the score is < 0.05, and tolerated if the score is 0/> 0.05.

**PolyPhen**: "Probably damaging" (it is believed most likely to affect protein function or structure), "Possibly damaging" (it is believed to affect protein function or structure), "Benign" (most likely lacking any phenotypic effect).

**PMUT**: NN output is the original output from the neural network for this mutation and its parameters. If that output is bigger than 0.5 it is predicted as pathogenic, otherwise as neutral.

**Figure 1:**
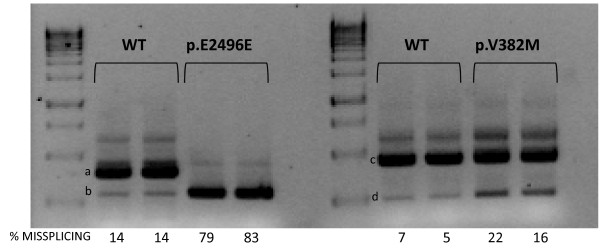
**In vitro splicing assays for p.E2496E and p.V382M mutations**. Gel electrophoresis shows the different splicing processes for WT minigene and mutants constructions. COS-7 cells transfection experiments were performed in duplicate. Numbers at the bottom of gels indicate the proportion (%) of misspliced transcripts compared to the full-length transcript. For the p.E2496E mutation, an evident increase of band b (corresponding to the aberrant transcript that only contains 37pb of the exon 40) can be observed with regard to the WT minigene expression product. For the p.V382M variant a small increase of the exon 7 skipping in the mutant minigene expression is observed (band d).

Four missense variants were classified as possibly-pathogenic (UV3). The variants p.R303H and p.Y1992C were described previously [https://grenada.lumc.nl/LOVD2/Usher_montpellier, [35]]. The novel change p.N3894D was not found in 200 control alleles and the segregation analysis proved that it co-segregates with the disease. However, only one program considered it as clearly pathogenic (See Table 6). The new p.V382M change, which affects the first base of exon 7, was not found in control samples and it was predicted to slightly affect splicing. The minigene assays only revealed a mild increase of the transcript excluding exon 7 (Figure 1, band d) when the variant was present, in comparison to the wild-type sequence.

Finally, six missense mutations were considered as pathogenic. p.C759F and p.C3267R were already described by others authors as damaging [https://grenada.lumc.nl/LOVD2/Usher_montpellier, [35]]. p.C3358Y and p.P4818L were classified by McGee et al. (2010) [30] and the LOVD-USH Database as likely-pathogenic (UV3). However, in the present study, p.C3358Y was detected in a patient together with another nucleotide change (p.C3267R) and the segregation analysis confirmed that the mutations were not in the same allele. p.P4818L was detected in a patient together with two other mutations that directly or indirectly cause a truncated protein (p.Q3368X and c.5278delG). The segregation analysis confirmed that the deletion and the nonsense mutation were in *cis *and the missense variant was in the other allele and cosegregated with the disease. The segregation analyses support the damaging effect of p.C3358Y and p.P4818L, so we have considered them as pathogenic. p.G44R and p.G3546R were novel; none of them was detected in 200 control alleles. The variant p.G44R was detected in a single patient and p.G3546R in three cases (one homozygous and two compound heterozygous cases); the results of the three computational analyses classified them as pathogenic (See Table 6).

### Silent variants

We also identified 20 silent variants, 17 were previously described as neutral [[36], https://grenada.lumc.nl/LOVD2/Usher_montpellier, [35]] and three were novel (See Table 3). Only the variant p.E2496E was categorized as pathogenic. It was not found in 200 control alleles and the segregation analysis confirmed that this mutation co-segregates with the disease. We detected this variant in *trans *in a patient who also had a premature stop codon. According to *in silico *analyses, it was predicted to create a *de novo *donor splice site (data not shown). The splicing alteration was confirmed using hybrid minigenes. The mutant construct generated a transcript that lacked the last 106 nucleotides of exon 40 (Figure 1, band b). This loss of nucleotides creates a new open reading frame, leading to a premature stop codon five amino acids downstream.

### Intronic variants

Fourty-six intronic variants located at non-canonical positions of splice sites, of which 20 are novel, were detected in the *USH2A *gene sequence. According to computational analysis, most of these novel variants were classified as possibly non-pathologic (UV2). (See Table 4).

## Discussion

In the present study, we have performed a wide mutational screening of the *USH2A *gene in 88 unrelated Spanish patients diagnosed with Usher syndrome. This analysis has led us to identify a total of 37 different pathogenic mutations, 23 of which had not been previously described: six nonsense, eleven deletions/insertions, two missense, three splice-site mutations and one isocoding variant. At least one mutation was identified in 43 cases and the two responsible mutations were detected in 25 patients (five homozygous and 20 compound heterozygous cases).

The genotype-phenotype correlation for those patients bearing two mutations is illustrated in Table 5. Most cases presented with classical USH2 clinical features. But, interestingly, in one patient (RP-259), the sensorineural hearing loss was profound, RP started at the age of 6 years and he also had vestibular dysfunction (clinical findings typical for USH1). In another intriguing case, phenotype manifestations started at the age of 50 years (RP-1703). We cannot discard the possibility that additional changes in *USH2A *or in other USH genes, present in these patients, have some modifying effect on the phenotype [37,38].

It is complicated to predict the consequences of missense, silent and intronic changes, in order to discriminate neutral variants from those with a pathogenic effect. We have used a number of bioinformatics' tools to predict the damaging effect of these variants. However, we must bear in mind that these results are only computing predictions and additional studies are necessary to confirm the effect of those changes not clearly classified. In this sense, i*n vitro *analyses for two variants located at non canonical splice sites which were predicted to affect the splicing (p.E2496E and p.V382M) showed that p.E2496E creates a *de novo *donor splice site stronger than the wild type site that leads to the loss of the last 106 nucleotides of exon 40. Thus, we have considered it as pathogenic. On the other hand, the presence of p.V382M revealed a mild increase of the transcript excluding exon 7 (Figure 1, band d) when the variant was present, but still, the normal transcript has a stronger expression. For this reason, this change has been classified as UV3.

The majority of mutations were found once or twice. Only the c.2299delG mutation was identified in more than 5 alleles. However, the cohort in our study is biased, because those patients in whom two mutations were detected in previous analyses (study of exon 13, exons 2-21 or microarray analyses) were not included in this work. Actually, the allele frequency of the c.2299delG mutation in the Spanish population is 15%, which is lower, in any case, than in other populations [39].

**Figure 2:**
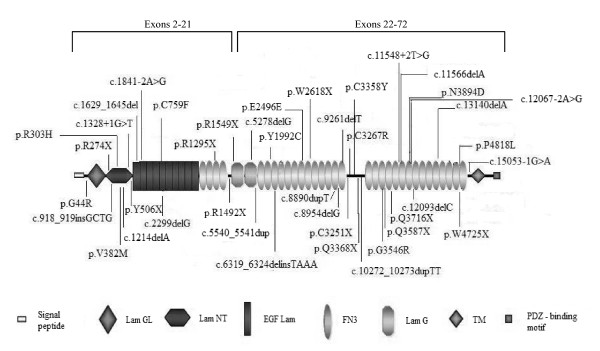
**Schematic illustration of the pathogenic and possibly pathogenic (UV3) mutations identified in this study along the USH2A protein domains**.

Figure 2 shows the distribution of all the pathogenic mutations detected in the present study, along the different domains of the USH2A protein. Mutations are located evenly throughout the protein and no "hot spots" were observed. Interestingly, there are two domains in which mutations are not detected: the transmembrane and intracellular domains. None of the studies performed in *USH2A *have detected mutations in the intracytoplasmatic region, involved in the interaction of the USH2A protein with harmonin and whirlin.

There are more than 160 pathogenic variants described in previous studies. Noteworthy, 23 mutations reported in this work are novel. If we compare the mutations detected in this study with those found in other Caucasian populations, only five mutations are common with the studies of Baux et al. (2007) [25] and McGee et al. (2010) [30] and three with the work of Dreyer et al. (2008) [28]. However, we can also find similarities with other populations, like non-Ashkenazi Jews. The splice-site variant c.12062-2A > G was detected in three patients, in homozygous state in one of them. This mutation was initially described by Auslender et al. (2008) [26], as one of the most *USH2A *prevalent mutations in non-Ashkenazi Jews. Later, it was also detected in the American population [30]. We do not know the origin of our three patients, but it is tempting to speculate that they are descendant of those Sephardic Jews that were expelled from Spain in 1492 [40].

We did not find any mutation in 45 families while in 18 the second mutation remained unidentified. The number of detected pathogenic variants is probably underestimated, because there may be mutations in regions which have not been analyzed (introns, 3' and 5' untranslated regions (UTRs), promoter region, distant enhancers...) or large insertions, deletions and rearrangements that cannot be detected with the conventional PCR techniques. Moreover, some of these patients may have mutations in other genes like *GPR98*, which seems to be responsible for approximately 3-6% of USH2 cases [41,42] or *DFNB31*, although the studies indicate a minor role of *DFNB31 *in USH2 [43,44]. Furthermore, USH1 genes may be responsible for phenotypically USH2 patients. Jaijo et al. (2010) [32] found two mutations in *CDH23 *in two patients diagnosed as USH2 and a high phenotypic heterogeneity due to *CDH23 *variants has been reported [45,46].

In this report, we have detected at least one mutation in 48.9% (43/88) of total patients. Considering only the patients clearly diagnosed with Usher syndrome type II, the mutation detection ratio raises to 56.9% (33/58). This detection rate is lower than expected because, as it has been mentioned before, the patient sample included in this study is biased. Thus, if we take into account all our USH2 patients studied so far (including results from previous studies [[19,9,23,32], unpublished data] and the present work), our database includes 102 typical USH2 patients with at least one mutation detected in the *USH2A *gene and 32 typical USH2 patients who have been studied for all exons of this gene and no mutation was found (Table 7). Thereby, our mutation detection rate rises considerably to 76.1% (102/134), making our percentage similar to those obtained by Baux et al. (2007) [25] and Dreyer *et al*. (2008) [28].

**Table 7:** Comparison between results obtained from the sample included in the present report with the global results for our total series.

	PRESENT REPORT SAMPLE				**TOTAL SERIES**^**#**^			
	
	0 MUT	1 MUT	2 MUT	1 + 2 MUT^*^	0 MUT	1 MUT	2 MUT	1 + 2 MUT^*^
**USH2**	43.1% (25/58)	22.4% (13/58)	34.5% (20/58)	56.9% (33/58)	23.9% (32/134)	16.4% (22/134)	59.7% (80/134)	76.1% (102/134)

**USHA**	54.5% (6/11)	18.2% (2/11)	27.3% (3/11)	45.5% (5/11)	33.3% (8/24)	25% (6/24)	41.6% (10/24)	66.7% (16/24)

**USHNC**	73.7% (14/19)	15.8% (3/19)	10.5% (2/19)	26.3% (5/19)	64% (16/25)	12% (3/25)	24% (6/25)	36% (9/25)

**TOTAL**	51.1% (45/88)	20.5% (18/88)	28.4% (25/88)	48.9% (43/88)	30.6% (56/183)	16.9% (31/183)	52.5% (96/183)	69.4% (127/183)

We consider as a mutation (MUT) only those clearly pathogenic. If we also consider UV3, the total figures in the present report sample are 51.1% (45/88); 17% (15/88); 31.9% (28/88) and 48.9% (43/88) for 0 MUT, 1 MUT, 2 MUT and 1 + 2 MUT respectively.

* Percentage of patients with at least one mutation in *USH2A*: patients with only one mutation + patients with 2 mutations (1MUT + 2MUT).

^# ^TOTAL SERIES represents the global results obtained from this, together with previous studies [19,9,23,32, unpublished data and present work]

## Competing interests

The authors declare that they have no competing interests.

## Authors' contributions

GG carried out the mutational screening of the *USH2A *gene in Usher syndrome patients, participated in the minigene constructions and expression and drafted the manuscript. MA carried out the mutational screening of the *USH2A *gene in controls and participated in the minigene constructions and expression. TJ carried out the Splice-site predictions and drafted the manuscript. RR carried out bioinformatics' predictions of the pathogenic effect of missense variations. AL participated in the mutational screening of the *USH2A *gene in Usher syndrome patients. AA and SB participated in patients' and controls' genomic DNA extraction. FB participated in clinical anamnestic data collection. RN, MB and CA participated in patient recruitment, pedigree and clinical anamnestic data collection. MD participated in the clinical diagnosis of patients. JM participated in patient recruitment, pedigree and clinical anamnestic data collection and drafted the manuscript. EA coordinated and supervised the study and drafted the manuscript. All authors read and approved the manuscript.
